# Supplementary material for: Are carbon emissions trading and green financial instruments synergistic? -Comprehensive quantitative research based on content analysis
Source: PLoS One. 2024 Mar 7;19(3):e0298601. doi: 10.1371/journal.pone.0298601 (PMC10919601; doi:10.1371/journal.pone.0298601)
Supplement: S1 Data — (ZIP) [file pone.0298601.s001.zip › Data/地级市Z_F工作报告.zip!/░▓╗╒/╨√│╟╩╨/└┤╫╘╣1⁄2╓┌║┼í╛┬φ┐╦╩2╛▌═°í┐_2010.docx]

过去五年工作

　　过去五年，是全市经济快速增长的五年。与“十五”末相比，生产总值、社会消费品零售总额、进出口总额翻一番；财政收入、地方财政收入、五年固定资产投资总量翻两番。首次实现主要经济指标增幅进入全省第一方阵、总量跻身全省第二方阵。完成了“十一五”节能减排目标任务。工业化进程不断加快。工业增速连续四年超过全省平均水平。农业产业化水平显著提高。服务业全面发展。文化产业发展迅速起步。金融业稳健发展。房地产业快速发展，投资和销售规模进入全省前列。县域经济增势强劲。在近五年全省综合考评和分类考核中，宁国连续三年位居综合十强县首位；广德连续两年进入“双十强”；郎溪连续两年位居二类县第一；绩溪连续两年位居三类县前两位。泾县、旌德位次不断前移。宁国、郎溪、绩溪连续两年获得全省“科学发展先进县”称号。

　　过去五年，是全市城乡面貌大为改观的五年。交通建设突飞猛进。累计完成公路、水运交通基础设施投资60亿元，是“十五”的近两倍，新改建公路里程5500公里，新开工建设高速公路220公里。升级改造了宣水、宣港、华龙、石千等一批干线公路。完成农村公路“村村通”4560公里。芜申运河宣城段进展顺利。成功争取四条高速铁路项目。水利、电力、通信等建设持续加强。城市建设力度不断加大。

　　过去五年，是全市改革开放不断深入的五年。国有集体企业改革基本完成。飞彩股份实现战略性重组，中鼎股份实现增发再融资。民营经济得到快速发展。国库集中支付、政府采购制度、乡财县管改革进一步完善。农村综合改革深入推进，粮食流通体制改革、集体林权制度改革、基层医药卫生体制改革顺利实施。文化体制改革重点任务顺利完成。新一轮政府机构改革和乡镇体制改革全面完成。市政公用事业特许经营市场化改革成效明显。大力支持金融和投融资体制改革。始终把招商引资作为经济工作的重要任务，招商引资总量和增幅位居全省前列，五年累计实际利用外资6.8亿美元，实际到位省外资金1544亿元，分别是“十五”期间的3.4倍和10倍。

　　过去五年，是全市民生持续改善的五年。与“十五”末相比，城市居民人均可支配收入、农民人均纯收入接近翻一番，年均分别增长13.7％和14.5％。城市居民收入水平在全省的位次明显上升，农民收入水平保持全省前列位次。认真组织实施省、市各项民生工程，累计投入资金39.5亿元。坚持开发式扶贫。切实加强就业工作。不断完善社保体系，社会保险五大险种参保人数连年超额完成省定目标。累计开工建设保障性廉租住房和拆迁安置房208万平方米，竣工105万平方米。城市人均住房面积达到30平方米。

　　过去五年，是全市社会事业全面发展的五年。科技对经济增长的贡献率逐步提高。初步建立统一规范的人力资源市场。组织实施中小学教育“六项工程”和“四个提升”计划。县市区全部建成合格职教中心，宣城职业技术学院新校区一期工程建成使用。广播电视“村村通”、乡镇综合文化站、农家书屋等工程取得新进展。圆满完成奥运火炬在宣城境内传递和迎亚运书法火炬火种采集任务，成功举办欢乐中国行、江淮情以及全国道德模范和身边好人现场交流会等大型活动。精神文明创建成果丰硕。全民健身活动深入开展。健全和完善了城乡公共卫生和医疗服务体系，新型农村合作医疗制度实现全覆盖。人口自然增长率始终保持在3.5‰以内。强化国土资源管理。深入推进“平安宣城”、“法治宣城”建设。人防建设取得长足进步。国防动员和后备力量建设得到加强。强化社会治安综合治理，社会保持和谐稳定。民族宗教，外事侨务、新闻出版、档案、地方志、气象、地震等工作取得新进步，妇女、未成年人、老年人、残疾人和红十字、慈善福利等事业实现新发展。

2010年工作

　　刚刚过去的2010年，全市经济社会发展延续了近年来的良好势头，增长速度进一步加快，质量效益进一步提高，发展后劲进一步增强，人民生活进一步改善。预计，全年实现生产总值515亿元，增长14.6％。财政收入84.7亿元，增长40.2％；地方财政收入49.8亿元，增长40.7％。固定资产投资730亿元，增长30％。规模工业增加值265亿元，增长27％。新增贷款102亿元，增长26.7％。社会消费品零售总额195亿元，增长19.2％。进出口总额6.7亿美元，增长43.8％。城市居民人均可支配收入15100元，增长13.5％；农民人均纯收入6200元，增长10.1％。

　　全力推进承接产业转移。完成了示范区建设空间规划和产业规划。出台了“四十条”促进政策。建立了重点产业、基础设施建设项目库。市开发区发展进一步加快，15平方公里的示范园区起步区开始建设。大力推进与国家电网、中建材等央企以及全国知名民营企业的发展合作，14个央企对接项目签约，总投资552亿元，其中5个项目开工建设；407个民企对接项目签约，总投资1559亿元，项目个数和总投资均居全省第二位，其中合同项目282个，居全省第一位，投资额800多亿元，居全省第四位。招商引资工作继续得到加强，新签省外投资项目1250个，实际到位省外资金700亿元，比上年增长1倍，总量位居全省第三，增幅位居全省第一；实际利用外资2亿美元，增长30％。跨省域合作共建园区项目达到6个。

　　大力优化经济结构。加强工业技改和重点项目建设，预计完成工业投资380亿元，增长39％。工业经济效益综合指数达到228.2，创历史最好水平。坚决淘汰落后产能。加快现代农业园区建设，推进种养业规模化、标准化，获得国家级标准化养殖示范小区4个。开展“食品工业推进年”活动。制定了加快服务业发展的政策措施，积极推进19个重点项目建设。深入开展“旅游服务设施建设年”和“旅游质量提升年”活动。

　　强力推进城乡基础设施建设。合福高铁宣城段开工建设，商杭、黄杭高铁和皖赣铁路等项目有序推进。继续实施水库除险加固，98处农村饮用水安全工程全部完工。加强了城市建设，累计投入近90亿元，其中市政基础设施投入25.3亿元。水阳江大道东南段及西北段、滨湖路、响山路延伸段、薰化路延伸段基本建成。九洲大道改造竣工通车。市区污水管网、宛溪河综合整治一期、敬亭山基础设施建设一期、梅溪公园主体工程等基本完工。环城大道、向阳大道、鳄鱼湖景区、北门棚户区改造等一批工程加快推进。

　　着力改善民生和发展社会事业。全面完成省定33项民生工程，累计投入资金15亿元。宣城中学新校区已经奠基，市奥体公园前期工作加速推进。竞技体育在省第十二届运动会上创历史最好成绩。成功举办全市第一届残疾人运动会。大力实施文化惠民工程。巩固“村村通”成果，广播、电视综合覆盖率达到97％以上。建立了基层医疗卫生机构公益性管理体制和新的运行机制。在全省率先建立了城市常住和流动人口户况管理平台。认真开展第六次人口普查。强化就业和社会保障工作，城镇新增就业人数、城镇养老保险参保人数完成任务情况名列全省前茅。新农保试点工作顺利实施，城乡低保和农村“五保”工作进一步加强。

过去五年经验

　　五年来，我们坚持以创新引领发展，致力于把上级精神与宣城实际紧密结合，创新发展理念和发展模式，创造性地开展工作，走出一条具有宣城特色的发展之路。

　　我们坚持以项目推动发展，把项目建设作为经济工作的重要抓手，千方百计扩大有效投入，不断增强经济发展后劲。

　　我们坚持以效能促进发展，深入开展民主考评活动，提高服务水平和质量，深化行政审批制度改革，完善经济运行调节、工作绩效考核等制度，不断优化发展环境。

　　我们坚持以良好的政治生态保障发展，把优化政治生态与提振精神状态紧密结合，充分调动各方面积极性和创造性，凝聚全市上下干事创业的强大合力。

未来五年发展目标·主要任务

　　未来五年，我们要坚持以科学发展为主题，以加快转变经济发展方式为主线，坚持工业化与城镇化、提升传统产业与发展新兴产业、扩大总量与提高质量、强市与富民、跨越发展与和谐发展“五个并重”，把宣城逐步建设成为皖苏浙交汇区域宜业宜游宜居的中心城市，面向长三角的新兴制造业基地、优质农产品供应基地、旅游休闲基地。

　　打造创新宣城。一是要以超常规的力度发展新材料等战略性新兴产业；二是要以超常规的力度提升汽车及零部件等传统产业；三是要以超常规的力度做大做强文化、旅游和农业等优势产业；四是以超常规的力度弥补服务业这一“短板”。为此，实施工业“5 3”工程和“千亿产业”计划，即提升汽车及零部件、机械电子、医药化工、新型建材，农副产品深加工五大传统产业，培育发展新材料、节能环保、现代装备制造三大战略性新兴产业。加强自主创新载体和服务平台建设，加快发展高新技术产业，力争产值达到400亿元，新增一批高新技术企业和创新型试点企业，建设一批国家和省级工程技术研究中心、企业技术中心。大力推动旅游、现代物流、金融、商贸流通、服务外包等生产性和生活性服务业加快发展，提高服务业比重和水平，打造全国知名的旅游目的地和商品集散地。全面提升农业产业化水平，加快建设家禽强市。着力发展规模农业、生态农业、观光农业。以发展食品工业为重点，努力打造家禽、林特产品两个超百亿产业集群，农产品加工产值达到600亿元。

　　打造文化宣城。我市文化底蕴十分深厚，要努力把文化资源转变为经济资源，文化优势转变为产业优势，促进文化事业全面繁荣和文化产业跨越发展。建成中国文房四宝产业园、中国茶府、瑞雅国际文化广场等一批重点文化产业项目，形成1－2个特色文化产业集群，创建6－10个具有较大影响的文化产业品牌，建设全国知名文化产品生产和交易基地。力争将文化产业培育成重要产业，文化产业增加值占GDP比重达到5％左右，综合实力位居省内同等城市前列，逐步把宣城建设成为“文化产业之城”。大力推进文化惠民工程，加强城乡文化基础设施建设，广泛开展群众性文化活动。建成市图书馆、奥体公园、城市规划展览馆和会展中心等，力争开工建设市科技馆、博物馆、美术馆和宣城大剧院等工程。

　　打造秀美宣城。加快中心城市建设，是“十二五”期间又一个突出的重点。要围绕建设宜业宜游宜居城市的目标，继续按照“做规划、拉框架、补功能、重管理、提品位”的思路和“通起来、亮起来、绿起来、活起来、雅起来”的要求，形成主城区“一城四片区”，即老城区服务业集聚区、东部交通物流新区、南部文化科技教育新区、西部新型工业新区、北部新型工业新区，以及彩金湖政务文化新区、南漪湖休闲运动度假新区的城市功能分区格局。中心城区建成“三环八射”道路网架，加快实施敬亭山、鳄鱼湖景区开发建设和北门棚户区改造；进一步加快宛溪河、泥河等市区五条河道的整治；启动水阳江、南漪湖综合治理和开发工程。加快高速铁路、高速公路、水运航道、区间快速通道及重要站场建设，进一步完善“村村通”公路工程。

　　打造和谐宣城。和谐发展是跨越发展的前提和保证。因此，必须要加强以改善民生为重点的社会建设，不断提升群众幸福指数。要通过增加就业岗位、提高最低工资标准、扩大经营性收入等渠道，提高城乡居民收入水平，力争五年后城市居民人均可支配收入、农民人均纯收入分别达到30000元左右和12500元左右。五年新增城镇就业12万人，城镇登记失业率控制在4.2％以内。实现基本养老保险、基本医疗保险城乡全覆盖，不断扩大失业、工伤、生育保险覆盖面。高度重视人才引进、培养和使用。巩固十五年基础教育成果，推进义务教育均衡发展。进一步加强公共卫生服务体系建设，努力实现人人享有基本医疗卫生服务的目标。人口自然增长率控制在3.5‰以内。加大保障性住房建设力度，完善住房保障体系。

　　通过五年的努力，力争实现“五个翻番”，达到“一个高于”、“一个同步”和“一个确保”，即生产总值、财政收入、规模工业增加值、服务业增加值、固定资产投资比2010年翻一番；主要经济指标增幅高于全省平均水平；城乡居民收入增长与经济发展速度保持同步；确保完成节能减排目标任务。到“十二五”末，生产总值力争突破1000亿元，年均增长15％左右；财政收入力争达到210亿元，年均增长20％左右；市、区两级财政收入占全市的比重超过三分之一；固定资产投资五年累计完成5000亿元以上；进出口总额20亿美元，年均增长27％左右，让宣城这座江南名城焕发出更加蓬勃的生机与活力。

2011年的重点工作

　　综合考虑，建议今年经济社会发展的主要预期目标是：生产总值增长13％左右；财政收入增长20％以上；固定资产投资增长20％以上；规模工业增加值增长25％以上；社会消费品零售总额增长19％左右；进出口总额增长27％左右；城乡居民收入增长与经济发展速度保持同步；节能降耗和污染减排控制在省定目标以内。

　　大力承接产业转移。加强园区建设。完善市开发区规划体系，优化西扩新区产业布局。开工建设日新西路等道路，推进大豪自来水厂、川气东输总输站、22万伏高压线路改造等工程建设。支持县市区开发区扩容升级。支持宁国开发区争创国家级开发区。大力推进合作共建园区建设。加强重点项目建设。力争完成工业投资400亿元左右。积极推进中鼎产业园、华明低速汽车、LED产业园、光伏产业园等项目建设。加强招商引资工作。改进招商方式，提高质量效益。围绕国家产业政策导向，以产业链构建为抓手，着力引进大型企业、相关配套项目和上下游项目，加快产业集群发展。加强节能减排工作。大力推广节能新技术。大力发展循环经济。实施节能改造、污染治理和废弃物综合利用，推进清洁生产。严格执行污染物排放标准和环境影响评价制度，严厉查处环境违法行为。深入推进城市环境综合整治。

　　加快发展现代服务业。着力做强文化产业。编制文化产业发展规划，完善产业发展布局，明确产业发展重点。扎实推进中国文房四宝产业园、宣纸文化园和中国茶府、瑞雅国际文化广场、宣酒文化园等项目建设。积极筹建国家文房四宝产品质量监督检验中心。加紧谋划徽派工艺产业园、宣城创意产业园、灵芝文化园等项目。支持新闻单位加快市场化改革。着力做强旅游业。积极推进皖南国际旅游文化示范区建设。完成南漪湖休闲运动度假新区规划编制和谢朓楼重建工作。启动新港国际城建设。启动5A级龙川景区和4A级敬亭山景区创建工作。力争全年接待国(境)内外游客750万人次，旅游总收入达到50亿元。着力做强金融业。积极引进金融单位来宣设立分支机构。做大做强地方法人金融实体，发展村镇银行，推动各金融机构加大有效信贷投入。着力做强商贸流通业。加快双桥物流园区建设，谋划九洲市场搬迁工作。

　　继续发展现代农业。提升农业产业化水平，扶持农业产业化龙头企业做大做强。推进现代农业示范区和农产品加工园区建设。继续推进农业标准化建设和“三品”认证工作。稳定粮食生产。加强“菜篮子”工程建设。推进市县两级农产品批发和零售市场建设。完善农产品质量体系建设，加强动植物重大疫病防控，确保农产品质量安全。规范并完善农村土地承包经营权流转市场。深化农村综合改革。力争集体林权制度配套改革实现新突破。健全农业保险制度，改善农村金融服务。加强新型农民培训。

　　大力改善城乡基础设施。确保市本级城市建设投入100亿元以上，其中市政基础设施投入25亿元以上。注重规划引领作用，加紧城乡统筹发展规划编制，完善城市公共设施等专项规划。加快环城大道、鳄鱼湖景区、北门改造等17个续建项目进度。开工建设宝城路、宣古路、宛溪河综合整治二期、城市规划展览馆及泥河、道岔河整治等26个重点项目。抓好500千伏广德输变电工程前期工作，推进220千伏军塘输变电等16项工程建设。加快城市中心变建设，完善城区配网网架。积极引导社会力量参与新农村建设。力争水阳江下游防洪治理等项目开工建设。大力实施城东联圩、青弋江灌区溪口枢纽和水库除险加固工程。认真组织实施农村饮用水安全工程。继续支持合福高铁宣城段加快建设。确保绩黄高速公路建成通车，加快宣宁、宁绩、芜雁、宁千和宣广高速公路宣城东、西出入口扩建等工程进度。确保溧广高速公路开工建设。

　　深入推进民生工程。认真组织实施省定33项民生工程。落实就业扶持政策，健全就业服务体系，多渠道增加就业岗位，鼓励自主创业。完善统一规范的人力资源市场。加强社会保障公共服务平台建设，进一步扩大社会保险覆盖面。继续做好新型农村养老保险试点工作。推进城乡低保规范化建设。加强农村敬老院建设，提高农村“五保户”集中供养率。开工建设廉租房、经济适用房、公共租赁房等保障性住房9万平方米。依法做好拆迁和老城区、棚户区改造工作。继续完善城乡医疗救助体系。全面落实强农支农惠农政策。加强扶贫开发工作。强化价格调控监管。

　　全面发展社会事业。完成市科技创业服务中心一期工程，推进合肥工业大学宣城研究院建设。深入实施中小学教育“四个提升”计划。实施市区中小学布局规划调整。加快推进市奥体公园、宣中新校区以及旌德和众职院项目建设。继续深化文化体制改革。抓好乡镇综合文化站和文化信息资源共享工程建设。促进广播影视事业的发展。做好非物质文化遗产的申报、保护和传承工作。开工建设市图书馆新馆。深化基层医药卫生体制综合改革。巩固完善新农合制度。强化重大疾病防控和药品安全风险预警机制。加强全国人口和计划生育综合改革示范市建设，稳定低生育水平。启动国家生态市和环保模范城市创建工作。强力推进节约集约用地，有序开展农村土地综合整治。继续发展老龄事业、残疾人事业、红十字事业和慈善福利事业。切实保障妇女和未成年人合法权益。支持工会、共青团、妇联等人民团体参与社会管理和公共服务。认真做好统计、民族宗教、外事侨务、地方志、防震减灾等工作。

　　切实维护社会和谐稳定。深入开展平安宣城建设。积极开展“六五”法制宣传教育。以群众工作统揽信访工作，积极畅通群众诉求渠道，依法规范信访秩序，加大解决信访突出问题的力度。继续推进安全生产“三项建设”和“三项行动”，强化事故源头管控和隐患排查治理，确保安全生产形势持续稳定。强化食品药品质量安全监管。加强应急指挥平台和应急救援队伍建设。完成国防动员机制与应急管理机制衔接试点工作。支持驻宣部队建设。加强国防教育、国防动员、人民防空和民兵预备役工作，继续开展“双拥”共建活动。

切实加强政府自身建设

　　加强政策研究，进一步增强决策力。坚持围绕发展抓谋划，认真学习研究政策，把握宏观政策机遇，深入基层调研，广纳民智民慧，力求站得高、看得远，想得全、谋得准。坚持围绕大局出思路，统筹协调解决带有普遍性、苗头性、倾向性的重大问题。坚持围绕难题求突破，创新思想观念和发展理念，探索行之有效的办法和措施，努力解决改革发展中的热点难点问题。

　　健全完善机制，进一步增强执行力。实干才是才干，落实才是务实。把心思放在干事业上，把功夫下到抓落实上，确定的事情就雷厉风行地干，部署的工作就一以贯之地抓。完善决策中的民主集中制、执行中的分工负责制、检查考评中的责任追究制。继续深化行政审批制度改革，健全民主考评活动长效机制，提高行政效能。加强政务督查、行政监察和绩效考核，抓住“不落实的事”，追究“不落实的人”。

　　做好群众工作，进一步增强凝聚力。始终站稳群众立场，坚持以人为本、执政为民。坚持深入群众，及时有效地消除隔阂、化解冲突、理顺情绪，努力保持政通人和的良好局面。更加重视社会建设和管理，大力发展社会公共事业，千方百计办好涉及群众切身利益的事，坚决不做任何损害群众利益的事，真正在思想上尊重群众、感情上贴近群众、工作上动员群众、生活上关心群众。

　　规范行政行为，进一步增强公信力。强化依法行政的意识，善于用法治思维和法律手段解决问题和矛盾。坚持依法、科学、民主决策，规范行政决策程序，完善行政决策风险评估机制，加强重大决策跟踪反馈。严格依法履行职责，完善行政执法体制和机制，规范行政执法行为。加强政府信息公开，全面推进办事公开制度，提高政务服务质量。加强行政复议工作。自觉接受市人大及其常委会的法律监督、政协的民主监督和司法监督。认真听取各民主党派、工商联、无党派人士及人民团体的意见，高度重视人民群众和新闻舆论监督。加强审计监督。

　　坚持清正廉洁，进一步增强约束力。政府工作人员特别是领导干部，要带头执行党风廉政建设各项制度和规定，做到大是大非能过硬、小事小节能过关。始终把正确行使权力当作重要使命和重大责任，切实为人民掌好权、用好权，树立清正廉洁的形象。认真开展领导干部作风建设专项学习教育活动，坚决纠正部门和行业不正之风，切实“治懒、治散、治庸、治贪”，努力在科学发展、全面转型的实践中创优争先、勤政廉政。
